# Proximate Composition, Antinutritional Content, Microbial Load, and Sensory Acceptability of Noodles Formulated from Moringa (*Moringa oleifera*) Leaf Powder and Wheat Flour Blend

**DOI:** 10.1155/2021/6689247

**Published:** 2021-03-31

**Authors:** Aemiro Tadesse Zula, Dagem Alemayehu Ayele, Woinshet Abera Egigayhu

**Affiliations:** ^1^School of Nutrition, Food Science and Technology Academic Center of Excellence in Human Nutrition, Hawassa University, Ethiopia; ^2^Center of Food Science and Nutrition, Addis Ababa University, Ethiopia

## Abstract

**Background:**

Noodle products are popular throughout the world, and they can be prepared from cereal like wheat, maize, and rice. Nowadays, healthy and nutritious product requirement has been increasing. Thus, research on the nutrition-rich but neglected crop is becoming visible nowadays to ensure global food security and to satisfy the nutritional need. Research indicated that moringa tree leaf powder has good nutritional value, but it is not yet customized and properly consumed.

**Method:**

The study is aimed at developing noodles from wheat flour and moringa leaf powder and evaluating proximate composition, antinutritional content (phytate and tannin), microbial load (total plate count and yeast and mold count), and sensory acceptability. The experiment contains four treatments and one control. The data from proximate composition, antinutritional content, microbial load, and sensory acceptability were subjected to SAS version 9 software. A complete randomized design was used to analyze the proximate composition, antinutritional content, and microbial load data, and a randomized complete block design was used to analyze the acceptability test.

**Result:**

The study revealed that in the noodles formulated from 80% durum wheat flour and 20% of moringa leaf powder, the ash, protein, fat, fiber, gross energy, phytate, and tannin content were increased by 39.39%, 10.86%, 153%, 42.2%, 3.43%, 39.83%, and 329.78%, respectively, as compared with noodles made from 100% durum wheat flour. However, moisture, total bacteria count, and yeast and mold count were decreased by 28.71%, 45.52%, and 55.93%, respectively. Similarly, the study also revealed that the acceptability test of noodles was decreased as moringa leaf powder concentration is increased.

**Conclusion:**

In conclusion, besides the good nutritional profile and antimicrobial capacity, moringa has antinutritional content and influences the sensory acceptability of products. Therefore, limiting the moringa leaf powder concentration is needed during the development of products using moringa leaf powder.

## 1. Introduction

Noodles are a staple food in several countries of the world which can be made from a range of flour including rice, wheat, maize, and potato using an extruder by adding water and flavorant like egg and spice [1]. Since noodles play a significant role in the nutritional and societal values of human beings, the consumers are highly demanding (Li M, 2012). Different kinds of noodles products exist in the world and can be made from durum wheat. According to Li et al. [2], the consumption of noodles has been increasing, but the nutritional composition is limited with low fat and protein content [3]. Therefore, there is a need to complement durum wheat with other raw materials like moringa leaf powder for enriching noodles with good nutritional value.

Moringa is an endemic plant in Ethiopia, and the plant is grown dominantly in the southern region around Konso, Gamo, Gofa, Sidamo, Dawro, and Konta. [4, 5] stated that the moringa plant has many vernacular names based on the area where it is grown, and it is named “Haleko” in Gamo, Gofa, Dawro, and Konta; “Shelagda” in the Konso language; and “Shiferaw” in Amharic. Saifu (2015) reported that the moringa tree leaf is inexpensive and used as famine food to ensure food security. According to NRC [6], besides the economic importance, the moringa tree leaf has a vital nutritional value which contains protein, amino acid, carbohydrate, vitamins, minerals, and organic acid. Oduro et al. [7] and Madukwe (2013) reported that the moringa tree leaf is also known for its antibacterial and anti-inflammatory activities.

The product development using moringa leaf powder is not yet customized and commercialized properly in developing countries like Ethiopia specifically Hawassa even though moringa provides multibenefit like solving malnutrition and food insecurity since malnutrition and food insecurity are visible in the developing country. Thus, the study is aimed at developing noodles from the blend of wheat flour and moringa (*Moringa oleifera*) leaf powder with different proportions and characterizing the proximate composition, antinutritional content, microbial load, and sensory acceptability.

## 2. Materials and Methods

### 2.1. Sources and Preparation of Raw Materials

The raw materials for the preparation of noodles were wheat flour and moringa leaf powder. Wheat flour and moringa leaf powder were obtained from Hawassa City in the Sidama regional state of Ethiopia. Both wheat flour and moringa leaf powder were sieved into fine flour of uniform particle size by passing them through a 0.5 mm mesh screen. The wheat flour and moringa powder were later packed by polyethylene bag and finally stored at room temperature (25°C). The wheat flour and moringa leaf powder were then mixed according to the formulation (Table 1).

### 2.2. Noodle Preparation

A pilot-scale standard laboratory instrument was used for noodle preparation. Composite flour from wheat flour and moringa leaf powder was made based on the blending proportions of 100 : 0, 95 : 5, 90 : 10, 85 : 15, and 80 : 20. The moringa leaf powder concentration is limited to 20% due to moringa having bitter flavor and slightly green color as reported in [8]. The composite flour was mixed using a small-scale benchtop electric dough mixer machine to achieve a homogenous mixture. After mixing the composite flour, distilled water was added and mixed continuously to ensure that the dough had adequate consistency for lamination. The prepared dough was divided manually into 200 g, laminated using home scale size lamination, and extruded using a small-scale laboratory use extruder. Finally, the extruded product (noodles) was cut into a similar size, dried, packed, and stored at room temperature (25°C) until it was cooked and served to the panelist for sensory evaluation.

### 2.3. Cooking Noodles

Noodle samples were cooked in a small stainless steel thick-bottomed saucepan for 7-10 min in salted boiling water at 95°C; occasionally, it was stirred with a wooden kitchen spoon to prevent sticking to each other, then strained, rinsed, and washed with cooled running water (20°C); before testing noodles, it was strained from cooking water and placed in a plastic bag for evaluation.

### 2.4. Chemical Composition Analysis

The proximate compositions of noodles were determined according to AOAC [9]. The moisture content was determined using the official method of 934.01, ash content was determined using the official method of 923.03, crude fat content was determined using the official method of 920.39, crude protein (*N* × 6.25) was determined using the official method of 981.10, crude fiber was determined by using AOAC [10], and total carbohydrate was determined by the difference method. Gross energy was calculated by using Atwater's conversion factor, 16.7 kJ/g for protein and carbohydrate and 37.4 kJ/g for fat. Condensed tannin and phytate contents were determined according to Norhaizan and Faizadatul (2009). The phytate content was calculated by dividing the measured value of phytic acid by molecular weight (240) of phytic acid.

### 2.5. Microbial Analysis of Noodles

Total bacteria and yeast and mold count were determined on noodle samples according to AOAC (1984). A gram of noodle samples was taken aseptically after storing for 2 days at room temperature (25°C) and homogenized in 99 ml sterile peptone water 0.1% in a blender for 2 minutes, and serial dilutions were made. Dilution of 0.1 ml was spread plated in sterile Petri dishes. The stomacher dilution representing 10^−1^, 10^−2^, 10^−3^, 10^−4^, and 10^−5^ was prepared using 9 ml peptone water tubes. The plate count agar (PCA) with chloramphenicol addition was used for yeast and mold count after incubation at 25°C for 5 days, and molten plate count agar (PCA) was used for total bacteria count after incubation at 35°C for 48 hrs. Visible colonies were counted using a colony counter, and the count was expressed as log CFU/g of the original sample.

### 2.6. Sensory Evaluation of Noodles

The panelists used in the study were Hawassa University Food Science students. The students used for the sensory evaluation took the course “Sensory evaluation and product development.” A total of 20 panelists participated in the study, and they were well trained regarding the experiment. The environment for sensory evaluation was controlled effectively, and the sensory evaluation took place in the panel box which included water for rinsing. Under the panel box, cooked noodles coded with three-digit numbers were randomly given to the panelists, and the sensory evaluation was carried out using a five-point hedonic scale (1 = dislike very much, 2 = dislike, 3 = neither like nor dislike, 4 = like, and 5 = like very much) for its color, taste, aroma, texture, and overall acceptability.

### 2.7. Experimental Design and Data Analysis

The data of proximate composition, antinutritional content, microbial load, and sensory acceptability of noodle samples from five treatments were subjected to SAS software version 9. Complete randomized design (CRD) was used to analyze proximate composition, antinutritional content, and microbial load, and randomized complete block design (RCBD) was used for analyzing the sensory score. The mean separation was done using Tukey's HSD test at *p* < 0.05.

## 3. Result and Discussion

### 3.1. Proximate Compositions

The proximate compositions of noodles made from composite flour of wheat and moringa leaf are presented in (Table 2). The moisture content of noodles was varied from 10.83 to 7.72%. Noodles made from 5, 10, 15, and 20% of moringa leaf powder had lower (*p* < 0.05) moisture content as compared to 100% wheat-based noodles. The study revealed that moisture content of noodles was decreased by 28.71% as moringa concentration is increased by 20%, and a comparable result was observed in noodles made from Anchote flour (*Coccinia abyssinica*) (10.41-5.57%) (Beruk, 2015). The lower moisture content might be due to the lower water absorption capacity of moringa leaf powder [11].

The protein content of noodles varied from 9.85 to 10.92%. The study revealed that noodles made with 15 and 20% of moringa leaf powder had higher (*p* < 0.05) protein content as compared to noodles made from 100% durum wheat flour and lower moringa leaf powder concentration (5 and 10%). From the study, the protein content was increased by 10.86% in noodles made with the blend of 20% moringa leaf powder; a similar study was found in [12].

The fat content of noodles varied from 0.49 to 1.24%. Noodles made from 20% of moringa leaf powder had higher (*p* < 0.05) fat content as compared to noodles made from 100% wheat flour and noodles made from 5, 10, and 15% of moringa leaf powder. The fat content was increased by 153% in noodles made from the blend of 20% moringa leaf powder; this showed that the fat content was increased as moringa leaf powder is increased. Since moringa had good nutritional profile as reported by Arise et al. [13], noodles made with a higher concentration of moringa leaf powder could have higher fat content.

The ash content of noodles was varied from 0.99 to 1.38%. Noodles made from 15 and 20% blend of moringa leaf powder had higher (*p* < 0.05) ash content as compared to noodles made from 100% wheat flour and noodles made with 5 and 10% blend of moringa leaf powder. The ash content was increased by 39.39% in noodles made from 20% blend of moringa leaf powder. The higher ash content in the noodles made from the blend of higher concentration of moringa leaf powder might be due to moringa leaf being known by its higher mineral content as reported in [14].

The fiber content of noodles varied from 1.54 to 2.19%. The study showed that fiber content is increased as moringa concentration is increased. Noodles made with the blend of 20% of moringa leaf powder had significantly higher (*p* < 0.05) fiber content, and the fiber content was increased by 42.2%. The higher fiber content of noodles toward the increase of the concentration of moringa leaf powder might be due to the higher fiber content of moringa leaf powder, and according to Madukwe et al. [15], higher fiber content is expected for very fine cereal.

The carbohydrate of noodles is varied from 76.3 to 77.37%. The carbohydrate content of noodles was not significantly different across the treatment, and this might be due to carbohydrate being determined by the difference method.

The gross energy is varied from 349.01 kcal to 361.01 kcal. The study showed that noodles made from 20% of moringa leaf powder had higher (*p* < 0.05) gross energy as compared to noodles made from 100% wheat flour and noodles made from the blend of 5, 10, and 15% of moringa leaf powder. The higher gross energy in the noodles made from the higher concentration of moringa leaf powder could be due to the higher protein and fat content.

### 3.2. Antinutritional Content

**Figure 1:**
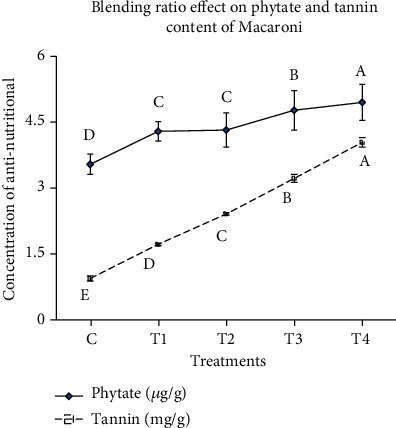
Effect of blending ratio on antinutritional content of wheat-moringa-based noodles.

Phytate and tannin content of noodles is shown in (Figure 1). The antinutritional (phytate and tannin) content of noodles was varied from 3.54 to 4.95 *μ*g/g and 0.94 to 4.04 mg/g, respectively. The study showed that antinutritional (phytate and tannin) contents of noodles were significantly increased as moringa concentration was increased from 5 to 20%. Similarly, noodles made with the blend of a higher concentration of moringa leaf powder had higher (*p* < 0.05) phytate and tannin content as compared to noodles made of 100% wheat flour. The higher phytate and tannin content of noodles made with the blend of higher concentration of moringa leaf powder could be due to the moringa leaf being known by its antinutritional content as reported by Ghasi et al. [16].

### 3.3. Microbial Load

**Figure 2:**
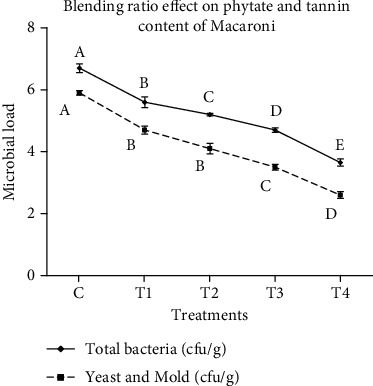
Effect of blending ratio on microbial load (total bacteria and mold and yeast) of wheat-moringa noodles.

The microbial load of noodles is shown in Figure 2. The total bacteria of noodles was varied from 6.7 to 3.65 cfu/g. Noodles made from 100% of wheat flour had higher (*p* < 0.05) total bacteria as compared to noodles made with the blend of moringa leaf powder. The study showed that the total bacteria of noodles was significantly decreased (*p* < 0.05) as moringa leaf powder concentration is increased from 5 to 20%. The lower total bacteria in noodles made with the blend of higher concentration of moringa leaf powder might be due to the antimicrobial activity of moringa leaf as reported in [17]. The microbiological standard of blended foods from 10^3^ to 10^5^ cfu/g is an acceptable value. The total plate count for cereal- and legume-based products exceeding 10^6^ cfu/g is considered microbiologically unsafe [18]. Noodles made from 100% wheat had higher total bacteria count as compared to the standard, and this might be due to personal hygiene as it is made first.

Both yeast count and mold count are also shown in Figure 2. The mean value for yeast and mold count of noodles was varied from 5.9 to 2.6 cfu/g. The study revealed that noodles made with the blend of 5 and 10% of moringa leaf powder had lower (*p* < 0.05) yeast and mold count as compared to 100% of wheat flour noodles. Contrary to this, 5 and 10% of moringa leaf powder-based noodles had higher (*p* < 0.05) yeast and mold count as compared to noodles made with the blend of 15 and 20% of moringa leaf powder. Therefore, like total bacteria, yeast and mold count was decreased as moringa concentration increased. The lower yeast and mold count for noodles made with the blend of higher concentration of moringa leaf powder might be due to antimicrobial activity of moringa as reported in [19].

### 3.4. Sensory Acceptability of Noodles

The sensory acceptability of wheat flour and moringa leaf powder-based noodles is presented in Table 3. The color, taste, aroma, texture, and overall acceptability score of noodles were varied from 4.91 to 3.17, 4.80 to 2.51, 4.85 to 2.87, 4.87 to 2.58, and 4.89 to 3.31, respectively. Noodles made from 100% wheat had higher (*p* < 0.05) sensory acceptability as compared to noodles made from 5, 10, 15, and 20% moringa. The study showed that the color, taste, aroma, texture, and overall acceptability of noodles were decreased significantly (*p* < 0.05) as moringa concentration is increased. The lower sensory acceptability might be due to moringa leaf powder being green in color, bitter taste, and not well adapted by consumer as people are adapted to normal color of noodles. According to [8], a moringa-supplemented product has bitter flavor and slightly green color which is not acceptable by the panel in terms of taste and aroma as the concentration increased. The lower texture might be due to moringa powder being made from the leaf and which is very fine, and a comparable study was found by Awobusuyi [20].

## 4. Conclusion

Proximate composition (ash, protein, fat, fiber, and gross energy) and antinutritional (phytate and tannin) content of noodles become high toward the increase of moringa leaf powder, but moisture and microbial load (total bacteria and mold and yeast count) were lowered as moringa leaf powder concentration increased. Except for noodles made from 100% wheat flour, all treatments were within the acceptable range of microbial load which is below 10^6^ cfu/g. Thus, using moringa leaf powder during product development could be good from the nutritional and microbial aspects. In contrary to this, the higher concentration of moringa leaf powder decreases the acceptability of sensory properties and increases the antinutritional content; thus, the concentration should be considered in maintaining the acceptance of sensory attributes.

## Figures and Tables

**Table 1:** Formulation of wheat flour and moringa leaf powder.

Composite flour	T1	T2	T3	T4	C
Wheat flour	95%	90%	85%	80%	100%
Moringa powder	5%	10%	15%	20%	—

T1 is 5% moringa and 95% wheat, T2 is 10% moringa and 90% wheat, T3 is 15% moringa and 85% wheat, T4 is 20% moringa and 80% wheat, and C is 100% wheat flour (control).

**Table 2:** Effect of blending ratio on proximate composition of wheat-moringa noodles.

Treatment	Moisture (%)	Protein (%)	Fat (%)	Ash (%)	Fiber (%)	Carbohydrate (%)	Energy (kcal)
C	10.83 ± 0.03^a^	9.85 ± 0.10^b^	0.49 ± 0.00^b^	0.99 ± 0.28^c^	1.54 ± 0.02^c^	76.3 ± 0.25^ab^	349.01 ± 0.25^c^
T1	8.78 ± 0.02^bc^	9.88 ± 0.03^b^	0.54 ± 0.37^b^	1.00 ± 0.29^c^	1.97 ± 0.01^bc^	77.23 ± 0.19^a^	353.3 ± 0.24^b^
T2	8.28 ± 0.12^bc^	9.72 ± 0.10^b^	0.48 ± 0.0^b^	1.20 ± 0.00^b^	1.85 ± 0.01^c^	77.37 ± 0.11^a^	355.08 ± 0.19^b^
T3	7.89 ± 0.09^c^	10.51 ± 0.14^a^	0.73 ± 0.35^b^	1.40 ± 0.29^a^	2.06 ± 0.27^b^	77.02 ± 0.28^ab^	356.69 ± 0.61^b^
T4	7.72 ± 0.04^c^	10.92 ± 0.40^a^	1.24 ± 0.35^a^	1.38 ± 0.28^a^	2.19 ± 0.28^a^	76.55 ± 0.31^ab^	361.01 ± 0.32^a^

C is 100 wheat (control); T1 is 5% moringa and 95% wheat; T2 is 10% moringa and 90% wheat; T3 is 15% moringa and 85% wheat; T4 is 20% moringa and 80% wheat. Means followed by different superscript letters across the column indicate significant difference at *p* < 0.05.

**Table 3:** Effect of blending ratio on sensory acceptability of wheat-moringa noodles.

Treatment	Color	Taste	Aroma	Texture	Overall acceptability
C	4.91 ± 0.38^a^	4.80 ± 0.52^a^	4.85 ± 0.45^a^	4.87 ± 0.54^a^	4.89 ± 0.37^a^
T1	4.31 ± 0.49^b^	4.57 ± 0.41^b^	3.96 ± 0.73^b^	3.91 ± 0.75^b^	4.14 ± 0.62^b^
T2	3.96 ± 0.22^c^	3.51 ± 0.55^c^	3.54 ± 0.52^bc^	3.72 ± 0.41^bc^	3.98 ± 0.56^bc^
T3	3.28 ± 0.35^d^	2.99 ± 0.38^d^	3.32 ± 0.32^c^	3.51 ± 0.52^d^	3.48 ± 0.49^d^
T4	3.17 ± 0.43^d^	2.51 ± 0.45^d^	2.87 ± 0.72^d^	2.58 ± 0.44^cd^	3.31 ± 0.34^d^

C is 100 wheat (control); T1 is 5% moringa and 95% wheat; T2 is 10% moringa and 90% wheat; T3 is 15% moringa and 85% wheat; T4 is 20% moringa and 80% wheat. Means followed by different superscript letters across the column indicate significant difference at *p* < 0.05.

## Data Availability

The data used and/or analyzed in the study are available from the corresponding author on reasonable request.
